# No changes in levels of bone formation and resorption markers following a broad-spectrum antibiotic course

**DOI:** 10.1186/s12902-018-0291-x

**Published:** 2018-09-04

**Authors:** Kristian H. Mikkelsen, Tina Vilsbøll, Jens J. Holst, Bolette Hartmann, Filip K. Knop, Morten Frost

**Affiliations:** 1 Steno Diabetes Center Copenhagen, Gentofte Hospital, University of Copenhagen, Hellerup, Denmark; 2 Department of Clinical Medicine, Faculty of Health and Medical Sciences, University of Copenhagen, Copenhagen, Denmark; 3 Department of Biomedical Sciences, Faculty of Health and Medical Sciences, University of Copenhagen, Copenhagen, Denmark; 4 The Novo Nordisk Foundation Center for Basic Metabolic Research, Faculty of Health and Medical Sciences, University of Copenhagen, Copenhagen, Denmark; 5 Endocrine Research Unit, University of Southern Denmark, Odense, Denmark; 6 Department of Endocrinology, Odense University Hospital, Odense C, Denmark

## Abstract

**Background:**

Intestinal bacteria influence bone remodeling in rodents, and antibiotic manipulation of the rodent gut microbiota increases bone formation and prevents ovariectomy-induced bone loss. In theory, these effects may be mediated by changes in sex hormone biotransformation in the gut, gut serotonin secretion or nutrition-induced secretion of glucagon-like peptide 2 (GLP-2) and glucose-dependent insulinotropic hormone (GIP). Antibiotics change the human gut microbiota, but the effect of antibiotic treatment on human bone turnover is unknown.

**Methods:**

We analyzed serum levels of bone turnover markers, serotonin, GLP-2 and sex hormones before, immediately after, and eight, 42 and 180 days after a 4-day per oral antibiotic cocktail (vancomycin 500 mg, gentamycin 40 mg and meropenem 500 mg once-daily) in twelve healthy adult males. Fasting and meal-stimulated procollagen type I amino-terminal propeptide (P1NP), C-telopeptide of type I collagen (CTX) and osteocalcin levels were measured.

**Results:**

While the antibiotic course reduced the stool abundance and composition of anaerobic bacteria as confirmed by cultivation studies, neither short nor long-term alterations in serum P1NP, CTX and osteocalcin were observed. Furthermore, we did not observe any changes in levels of serum GLP-2, serotonin or sex hormones.

**Conclusion:**

Eradication of anaerobic bacteria from healthy adult males had no effect on serum bone turnover markers.

## Background

Bone remodeling involves a coordinated interplay of osteoblastic and osteoclastic activity. Recent evidence from animal studies suggests that intestinal microorganisms (i.e. the gut microbiota) and in particular gut bacteria, may influence bone remodeling, but results of animal studies are conflicting. Thus, 7–9 weeks old mice (C57BL/6) raised under germ-free conditions had reduced number of osteoclasts and increased bone mineral density (BMD) compared to conventionally raised animals, and re-colonization with gut bacteria normalized these features [1], indicating that microbiota inhibit osteoclastogenesis and bone metabolism. By contrast, presence of microbiota was associated with increased BMD and femoral length in 8 weeks old mice of a different strain (BALB/c) [2]. Furthermore, colonization of 2 months old germ-free mice (CB6F1) with gut microbiota caused a transient decrease in bone formation, which was followed by an increase in bone formation with greater longitudinal and radial bone growth at ten months of age [3]. Microbiota decreased trabecular bone volume fraction and increased levels of osteoclastic cytokines in 20 weeks old mice (C57BL/6) with leuprolide-induced estrogen deficiency but not in controls [4], and minocycline, a broad-spectrum tetracycline antibiotic, increased BMD and bone formation rate in ovariectomized 22–24 months old rats (Wistar) [5], indicating that the effect of microbiota on bone metabolism may depend on sex hormone levels.

Gut bacteria have the potential to influence bone metabolism through at least five different pathways: 1) By modulating secretion of gut-derived serotonin and/or gut hormones, which are involved in postprandial bone turnover [6, 7], 2) by chancing host immune cell activity with subsequent impact on bone cell differentiation and activity [1, 8], 3) by metabolizing and transforming intestinally excreted steroid compounds, including sex hormones [9], 4) by stimulating activity in the somatotropic axis [2, 3], and 5) by influencing intestinal absorption of vitamins, calcium or other micronutrients involved in bone homeostasis [10].

Antibiotic treatment results in profound short-term changes of the gut microbiota and several kinds of antibiotics have been shown to alter the rodent bone homeostasis: Low-dose penicillin, vancomycin and chlortetracycline all increased BMD in 3 week old but not 7 week old mice (C57BL/6) [11], and low-dose penicillin increased bone mineral content (BMC) and BMD in female but reduced BMC in male 20 week old mice (C57BL/6) [12], suggesting that the effect of microbiota varies with the skeletal development and between sexes. Moreover, broad-spectrum antibiotics, by changing the gut microbiota, decreased insulin-like growth factor 1 (IGF-1), increased bone mass and reduced bone resorption in two-month-old BALB/c mice [3] and broad-spectrum antibiotic treatment increased the expression of incretin hormones including glucagon-like peptide 2 (GLP-2) [13], which in turn may influence bone resorption [14], and decrease the inflammatory state in rodents [15].

The potential effects of antibiotic-induced changes in gut bacteria composition on bone metabolism have never been studied in humans. We hypothesized that changes in gut bacteria composition would link to bone metabolism, and in order to investigate underlying mechanisms we analyzed serum/plasma levels of serotonin, sex hormones, GLP-2 and bone turnover markers before and after a four-day, broad-spectrum antibiotic course in healthy adult males.

## Methods

### Study participants and experimental procedures

This investigation was designed to assess effects of changes in gut microbiota on glucose homeostasis and bone metabolism assessed using the biochemical markers of bone turnover: serum procollagen type I amino-terminal propeptide, C-telopeptide of type 1 collagen and osteocalcin. Data regarding glucose metabolism, high-sensitivity C-reactive protein (hs-CRP), gut and pancreatic hormone secretion have been published previously, including data on glucose-dependent insulinotropic polypeptide (GIP) and information about the protocol and experimental procedures [16]. In short, blood samples were taken from 12 healthy, male volunteers subjected to a four-day broad spectrum antibiotics course consisting of once-daily administration of 500 mg meropenem, 500 mg vancomycin and 40 mg gentamicin dissolved in apple juice and ingested orally. The combination of these three non-absorbable antibiotics was chosen to eradicate as many gut bacteria as possible, with the lowest possible risk of side-effects. The participants (age (mean value with 95% confidence interval (CI) in brackets): 23.4 (20.0–26.4) years; body mass index (BMI): 22.6 (21.3–23.8) kg/m^2^ were examined after an overnight fast on five study visits: before (day 0), immediately after (day 4) and following 8, 42 and 180 days after initiation of the antibiotic course. On three of these visits (day 0, day 4 and day 42), 4-h standardized liquid meal tests were performed. Blood samples were drawn 30 and 0 min before and 15, 30, 45, 45, 60, 90, 120, 240 min after ingestion of a liquid mixed meal (2205 kJ, 64.4 g carbohydrate, 20.3 g fat and 21.0 g protein, Nutridrink, Nutricia, Allerød, Denmark). Plasma/serum levels of GLP-2 and bone turnover markers were determined during fasting and postprandial conditions at these visits. On all visits, plasma/serum levels of serotonin, sex hormones and bone turnover markers were determined during fasting conditions. Stool samples were collected prior to each of the five visits for cultivation-based assessment of stool bacteria abundances.

### Laboratory methods

All blood samples were drawn into ice-cooled 10 ml vials without anticoagulants, except for blood samples taken during meal tests on day 0, 4 and 42 for the analysis of GLP-2 and bone turnover marker levels (which were collected in EDTA-vials and serum vials with clot inhibitor, respectively). Following minimum 15 min of coagulation, blood was centrifuged and serum aliquots were stored at − 80 °C until analysis. Serum procollagen type I amino-terminal propeptide, C-telopeptide of type 1 collagen and osteocalcin were measured with a chemiluminescence method using an automated immunoassay system (iSYS, Immunodiagnostic Systems Ltd., Boldon, England). Serum serotonin was measured using an ELISA kit for research laboratories (Fitzgerald, Acton, USA). Serum levels of testosterone, estradiol (total) and estrone sulfate were measured by high-pressure liquid chromatography with tandem mass spectrometry detection (AB Sciex API 6500, Framingham, USA). Serum levels of sex hormone-binding globulin were determined by a sandwich chemiluminescence assay (Roche Cobas 6000 analyzer, Rotkreuz, Switzerland). Plasma concentrations of GLP-2 were measured using a specific radioimmunoassay for the intact, active hormone (code no. 92160), as previously described [17].

### Calculations and statistical analysis

Statistical analyses were carried out using Prism 6 for Windows (Graph Pad, California, USA). The repeated measures analysis of variance model was used to compare differences between day 0 (baseline) value and day 4, 8, 42 and 180 values, correcting for multiple comparisons by use of the Dunnett’s test. Gaussian distribution and sphericity were assumed in all calculations. Postprandial excursions of bone turnover markers and GLP-2 were summarized into area under the curve (AUC) values, calculated using the trapezoidal rule. Results are reported as means and 95% confidence intervals (95% CI) unless otherwise stated.

## Results

A twenty-fold reduction in stool anaerobic (total) gut bacteria abundance and a reduction in coliform, enterococci and bifido bacteria abundances to below the detection limit were noted from day 0 to day 4 as previously reported [16]. Stool abundance of aerobic bacteria increased slightly immediately after the antibiotics course, but no long-term changes in the stool abundances of the five groups of gut bacteria were observed.

**Fig. 1:**
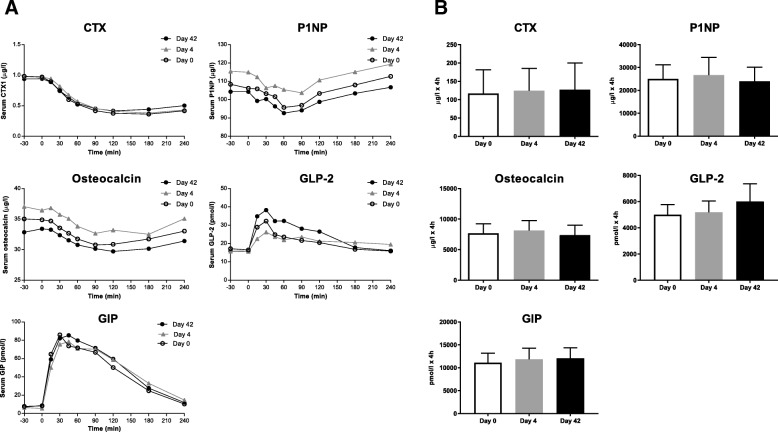
**a**. Serum levels of C-telopeptide of type I collagen (CTX), procollagen type I amino-terminal propeptide (PINP), osteocalcin and plasma levels of glucagon-like peptide 2 (GLP-2) and glucose-dependent insulinotropic polypeptide (GIP) during fasting and postprandial conditions before (day 0), immediately after (day 4) and six weeks after (day 42) the broad-spectrum antibiotic cocktail. GIP results have been previously published [16]. **b**. Postprandial excursions of bone turnover markers, GLP-2 and GIP summarized into area under the curve (AUC) values, reported as mean ± 95% confidence intervals. No statistically significant changes were observed in GLP-2, GIP or the bone turnover markers when comparing the area under the curve at day 0 with that of day 4 or day 42

On all study days, fasting serum levels of bone turnover markers were within the expected range (Table 1). Serum levels of bone turnover markers were reduced by consumption of food (Fig. 1), but the antibiotic treatment had no effect on fasting and postprandial levels of bone turnover markers (Table 1, Fig. 1).

**Table 1:** Serum levels of procollagen type I amino-terminal propeptide (PINP), osteocalcin and C-telopeptide of type I collagen (CTX), testosterone, estradiol, estrone sulfate, sex hormone-binding globulin (SHBG) and serotonin measured in the fasting state on each of the study days

	Day 0		Day 4		Day 8		Day 42		Day 180	
P1NP (ug/l)	108	(81–136)	116	(81–150)	114	(76–152)	104	(75–134)	113	(84–142)
Osteocalcin (ug/l)	35	(28–42)	37	(30–45)	36	(28–43)	33	(26–40)	35	(25–45)
CTX-1 (ug/l)	0.98	(0.68–1.29)	0.98	(0.72–1.24)	0.91	(0.64–1.17)	0.94	(0.67–1.21)	0.87	(0.58–1.15)
Testosterone (nmol/l)	19	(16–22)	21	(17–25)	23	(19–27)	21	(18–24)	26	(16–35)
Estradiol (pmol/l)	93	(75–112)	91	(68–114)	83	(63–103)	90	(70–110)	88	(59–117)
Estrone sulfate (umol/l)	19.7	(11.8–27.6)	15.7	(97.3–21.7)	22.1	(10.0–34.2)	20.3	(81.6–32.5)	22.8	(12.7–32.9)
SHBG (nmol/l)	34	(22–46)	36	(24–48)	40†	(28–52)	34	(23–45)	36	(26–46)
Serotonin (ng/ml)	164	(92–236)	178	(95–261)	199	(116–282)	180	(101–258)	221	(139–303)

Data are expressed as mean with 95% confidence intervals in brackets. † denotes *p* < 0.05 (following correction for multiple comparisons), indicating a significant change compared to day 0

None of the participants had serum levels of total testosterone below the normal range for healthy men in the same age group [18]. Overall, serum levels of sex hormones and serotonin were unaffected by the antibiotic course, but a small and temporary increase in sex hormone-binding globulin was seen from day 0 (34 nmol/l (95% CI 22–46)) until day 8 (40 nmol/l (95% CI 28–52)) (Table 1). Plasma GLP-2 increased after meal intake but there was no difference in the AUC on day 0, 4 and 42 (Fig. 1b). Similar, as reported earlier [1], the GIP responses to meal intake were not affected by the antibiotic treatment (Fig. 1b) and hs-CRP levels were below 3 mg/l at all time points (Table 1) [16].

## Discussion

To our knowledge, this is the first report on potential effects of broad-spectrum antibiotics on markers of turnover in humans. In order to reduce the risk of overlooking relevant protracted changes in the investigated variables, assessments of our participants were made 42 and 180 days after the antibiotics course. Despite a substantial short-term reduction in anaerobic gut bacteria abundance in the present human study, neither short nor long-term changes were seen in fasting or postprandial levels of bone turnover markers. Additionally, we investigated whether alterations in gut microbiota influenced serum levels of sex hormones and serotonin, and plasma levels of GLP-2 and GIP. While subtle and clinically irrelevant changes in sex hormone-binding globulin were observed, changes in levels of serotonin, GLP-2 and GIP were not observed, suggesting that short-term exposure to broad-spectrum antibiotics has no effect on sex hormones, serotonin, GLP-2 and GIP secretion.

Preclinical data on the effect of the gut microbiota on bone metabolism are conflicting, with reports suggesting either deleterious or beneficial effects of the gut microbiota on bone metabolism [1–3], and antibiotics-induced alterations in the composition of the microbiota caused a temporary increase in BMD [11] or diverse effects in male and female eugonadal mice [2, 12].

The study population comprised young, healthy men with no history of metabolic bone disease at time of peak bone mass. The fasting levels of bone turnover markers as well as changes in bone turnover markers during food intake were similar to those previously published [14], supporting that bone metabolism in the study population was normal. However, we did not observe any effect of a combination of short-term broad-spectrum antibiotics on bone metabolism assessed using biochemical markers. Based on the available evidence from rodent models it might be hypothesized that antibiotic exposure in early life or in adulthood could influence bone metabolism in humans. In a population-based cohort study of Finnish children there was a positive association between exposure to antibiotics in infancy and BMI and height at 24 months of age [19], but antibiotics exposure later in life was not associated with an increased risk of fractures in men in a Danish register-based study that comprised more than 15,000 fracture cases and 47,000 controls [20].

There is some evidence from rodent models, that manipulation of the gut microbiota may affect plasma levels of sex steroids [9] and that bacterial enzymes may facilitate intestinal transformation of estrone to estradiol and deconjugation of sex steroids [21]. Antibiotic treatment has previously been shown to increase fecal excretion of conjugated estrogens in both men and women, although with no or only limited impact on plasma sex hormone levels [22]. In line with the latter observation, we did not see any significant changes in serum levels of total testosterone, estradiol or estrone sulphate following the antibiotic course in this study. The link between low-grade inflammation and bone loss is well-established [23], and antibiotic eradication of inflammatory gut bacteria could theoretically lead to increases in bone formation and lower bone resorption by lowering endotoxemia [24]. However, the preset study was not designed to investigate effects of antibiotic treatment on inflammation as hs-CRP levels were below 3 mg/l at all study points.

This study has a number of important limitations that should be acknowledged. First, the limited number of participants and the substantial biological variability in bone turnover markers caused by diurnal rhythms, changes in diet etc. [14] means that we may have overlooked changes in levels of bone turnover markers caused by the antibiotic treatment. Second, the limited number of participants and the large number of statistical tests resulting from comparison of baseline and subsequent measurements result in a high risk of chance findings. In order to reduce the risk of type 1 errors, Dunnett’s test for multiple comparisons was used to evaluate the possible changes with time. Third, surrogate markers of bone turnover were used in the present investigation, and we cannot exclude the possibility that direct assessment of bone remodeling by dynamic bone histomorphometry could have shown effects of changes in gut microbiota on bone metabolism. Fourth, the absence of changes in bone turnover markers in our study could relate to the short-term antibiotics exposure or the study of a metabolically healthy adult male population free of bone disease. Our findings do not exclude that relevant changes in bone turnover markers may occur following treatment with other types of antibiotics, longer exposure time or in other study populations. Prominent changes in rodent bone turnover have been seen following gut microbiota manipulation in early life [11, 12] or during sex steroid deficiency [4, 5]. It would therefore be of interest to investigate the effects of antibiotic treatment on early life bone development or bone turnover in post-menopausal women.

## Conclusion

Eradication of anaerobic gut bacteria in healthy adult males had no significant short or long-term effect on serum levels of bone turnover markers, serotonin, sex hormones or GLP-2 in healthy non-diabetic men.

## Abbreviations

AUC: Area under the curve
BMC: Bone mineral content
BMD: Bone mineral density
BMI: Body mass index
CI: Confidence interval
GIP: Glucose-dependent insulinotropic polypeptide
GLP-2: Glucagon-like peptide 2
Hs-CRP: High-sensitivity C-reactive protein
IGF-1: Insulin-like growth factor 1
